# Spatio-Temporal Variations of High and Low Nucleic Acid Content Bacteria in an Exorheic River

**DOI:** 10.1371/journal.pone.0153678

**Published:** 2016-04-15

**Authors:** Jie Liu, Zhenyu Hao, Lili Ma, Yurui Ji, Mark Bartlam, Yingying Wang

**Affiliations:** 1 Key Laboratory of Pollution Processes and Environmental Criteria (Ministry of Education), College of Environmental Science and Engineering, Nankai University, Tianjin, China; 2 Tianjin Key Laboratory of Environmental Remediation and Pollution Control, College of Environmental Science and Engineering, Nankai University, Tianjin, China; 3 State Key Laboratory of Medicinal Chemical Biology, Nankai University, Tianjin, China; 4 College of Life Sciences, Nankai University, Tianjin, China; 5 State Environmental Protection Key Laboratory of Microorganism Application and Risk Control, Graduate School at Shenzhen, Tsinghua University, Shenzhen, Guangdong, China; 6 College of Chemistry and Chemical Engineering, Southwest Petroleum University, Chengdu, Sichuan, China; Sun Yat-Sen University, CHINA

## Abstract

Bacteria with high nucleic acid (HNA) and low nucleic acid (LNA) content are commonly observed in aquatic environments. To date, limited knowledge is available on their temporal and spatial variations in freshwater environments. Here an investigation of HNA and LNA bacterial abundance and their flow cytometric characteristics was conducted in an exorheic river (Haihe River, Northern China) over a one year period covering September (autumn) 2011, December (winter) 2011, April (spring) 2012, and July (summer) 2012. The results showed that LNA and HNA bacteria contributed similarly to the total bacterial abundance on both the spatial and temporal scale. The variability of HNA on abundance, fluorescence intensity (FL1) and side scatter (SSC) were more sensitive to environmental factors than that of LNA bacteria. Meanwhile, the relative distance of SSC between HNA and LNA was more variable than that of FL1. Multivariate analysis further demonstrated that the influence of geographical distance (reflected by the salinity gradient along river to ocean) and temporal changes (as temperature variation due to seasonal succession) on the patterns of LNA and HNA were stronger than the effects of nutrient conditions. Furthermore, the results demonstrated that the distribution of LNA and HNA bacteria, including the abundance, FL1 and SSC, was controlled by different variables. The results suggested that LNA and HNA bacteria might play different ecological roles in the exorheic river.

## Introduction

In natural aquatic environments, planktonic bacteria tend to cluster into two distinct subgroups, namely high nucleic acid content (HNA) bacteria and low nucleic acid content (LNA) bacteria, by flow cytometry (FCM) measurement in combination with nucleic acid staining [1]. This classification based on cellular size and fluorescence intensity of bacteria is widely observed in marine environments [2–6]. When first observed by FCM, LNA bacteria were regarded as inactive, dead or dying cells [7,8]. However, recent studies demonstrated that LNA bacteria were metabolically active [1,5,9]. It was found that LNA bacteria can survive and grow in oligotrophic environments due to their high affinity and binding-protein dependent uptake system [10]. Meanwhile, certain special cellular membrane constitutions could protect LNA bacteria from oxidation [11]. It was reported that LNA bacteria could adopt a dormancy strategy to overcome unfavorable environmental conditions [12].

In terms of ecological function, LNA bacteria were reported to play at least an equal niche role as HNA bacteria in the ecosystems [9,10,13–15]. Hence, information on the relation and variance between LNA and HNA bacteria are important to a better understanding of these two widespread groups of bacterioplankton in aquatic environments, and provide important foundation for further exploration of their environmental applications. The data on LNA and HNA bacterial abundance and activities have mainly come from marine environments [2,3]. It was found that the abundance and flow cytometric characteristics of LNA and HNA bacteria were strongly regulated by environmental variables, e.g. temperature, salinity, chlorophyll-α and nutrient conditions [5,6,16,17]. However, whether their variations on abundance and cytometric characteristics have similar pattern and how the variations respond to environmental factors are still unclear.

The distribution of LNA and HNA bacteria was found to change seasonally [18,19]. It was reported that although the growth rate of HNA bacteria in Lake Biwa generally exceeded that of LNA bacteria, LNA bacteria grew faster than and were grazed as fast as HNA bacteria in late August, when nutrients were severely limited [20]. Gomes and colleagues reported that HNA bacteria were apparently more responsive to the winter-spring phytoplankton bloom than LNA bacteria [21]. Nevertheless, to the best of our knowledge, information on the distribution and variations of LNA and HNA bacteria in freshwater environments is very limited, especially for the gradient along the river to the ocean.

The aims of the present study were 1) to analyze the temporal and spatial variations of the LNA and HNA bacterial abundance and cytometric characteristics along an exorheic river (the Haihe River); and 2) to estimate the effects of the driving factors on the distribution pattern of these two subgroups by multivariate analysis.

## Materials and Methods

### Ethics Statement

Water samples were taken from the Haihe River, where no specific permission is required. The Haihe River is a public river running through Tianjin, China. It is not privately owned or protected. The current study did not involve endangered or protected species. Water sampling procedures were reviewed and followed the Chinese Standard of Collection and Preservation of Water Samples (GB/T 5750.2–2006).

### Sampling sites

**Fig 1:**
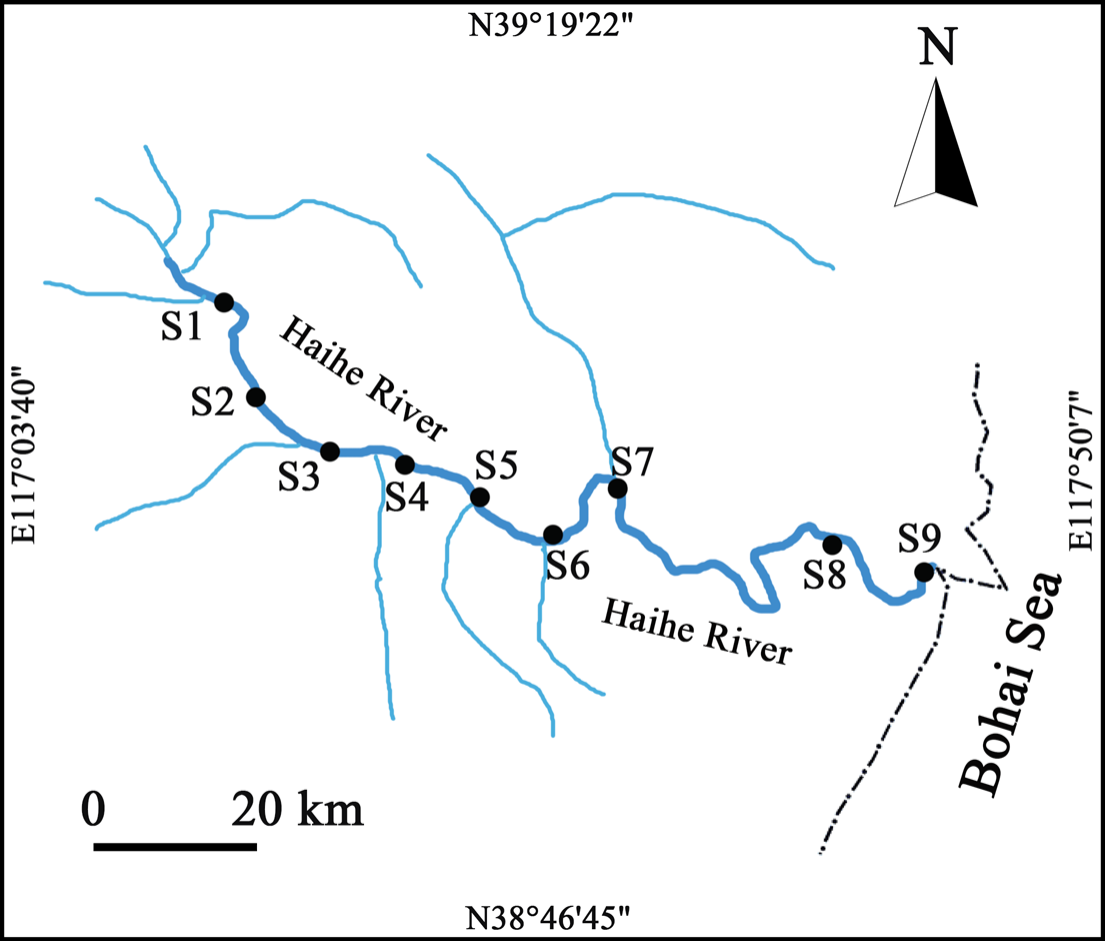
Sampling sites in the Haihe River. Black solid circles (●) indicate the sampling stations, and the black dash-dot curve (—·—) and blue solid curve (**━**) indicate the coastline and river, respectively.

The samples were collected at nine sampling stations along the Haihe river, which inflows into the Bohai Sea and is the largest river in Northern China (Fig 1) [22]. Water samples were collected from each station in four seasons: the autumn (September, 2011), winter (December, 2011), spring (April, 2012), and summer (July, 2012). At each sampling station, 2 L water samples were collected in clean and sterile bottles from a depth of about 0.5 m below the water surface. Samples were stored at 4°C during transportation and processed immediately within 24 hours after sampling.

### Flow cytometry analysis

Flow cytometry analysis was performed as described in Ma et al. [22]. One milliliter water sample was stained with 10 μL/ml SYBR Green I (1:100 dilution in dimethyl sulfoxide as the working solution; Invitrogen, USA), and incubated in the dark for 15 min at room temperature before measurement. The FCM (CyFlow Space instrument, Partec, Germany) specific instrumental gain parameters settings were as follows: SSC = 369 (log3), FL1 = 380 (log4), FL3 = 750 (log4). Bacterial communities were gated through the two-parameter dot-plot of green fluorescence (FL1) and side scatter (SSC), then the LNA and HNA bacterial concentrations were counted separately and the respective geometrical means of FL1 and SSC of LNA and HNA were calculated. All samples were measured in triplicate. Water samples were diluted in Milli-Q water (cell-free) so that the bacterial concentration was always less than 2×10^5^ cells/mL during the FCM measurement. The instrument detection limit was below 500 cells/s with an average standard deviation of 5%.

### Water environmental parameters

The temperature, pH and salinity of sampling waters were measured using YSI EC300 Water Quality Sonde. Water physical and chemical properties e.g. total suspended solids (TSS), total nitrogen (TN), nitrate (NO_3_), total phosphorus (TP), total dissolved phosphorus (TDP), total organic carbon (TOC), and chlorophyll-α (Chl-a) were measured as described in our previous studies [22, 23].

### Statistical Analysis

Analysis of variance (ANOVA) was conducted to test the significance of differences in measured or calculated parameters by using R statistical software (http://www.r-project.org/). A multivariate redundancy analysis (RDA) was performed by Canoco software (Canoco for Windows version 4.5) to further illustrate the changes of LNA and HNA bacteria in response to environmental factors [24]. The data were centered and standardized before redundancy analysis, and the Monte Carlo test was used to examine the significance of the RDA method. Meanwhile, a generalized linear model (GLM) and generalized additive model (GAM) were performed in a stepwise manner to predict the LNA and HNA bacterial abundance and flow cytometric characteristic response to environmental ordination axes in RDA. The axes were constrained by environmental variables, and the visualization formula constructed in terms of linear, quadratic or cubic degrees of GLM, then F statistics were used to test the significance in both GLM and GAM [25].

## Results and Discussion

### Temporal and spatial variations of LNA and HNA bacterial abundance and FCM characteristics

**Fig 2:**
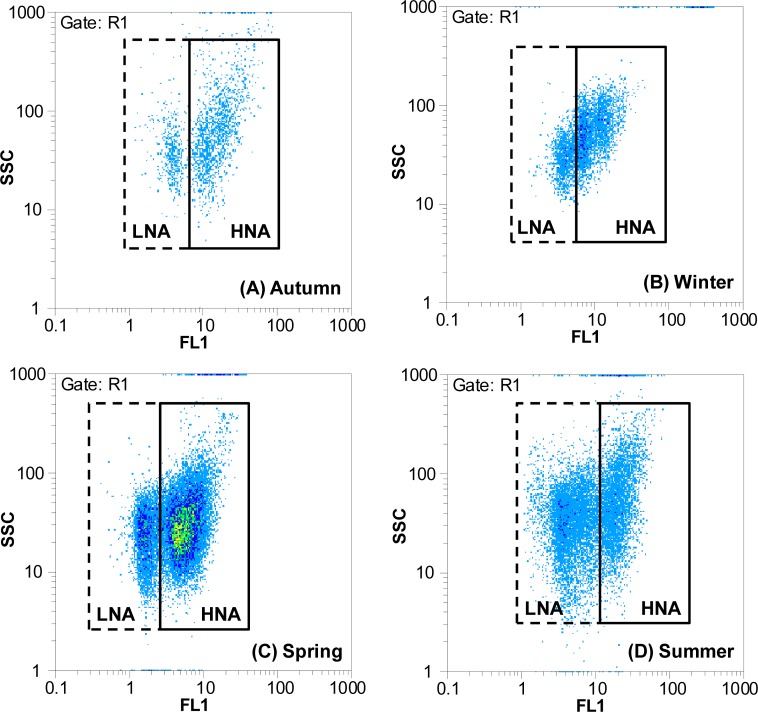
Flow cytogram example of LNA and HNA bacteria in the Haihe River. Dashed lines indicate LNA bacteria, and solid lines indicate HNA bacteria.

**Fig 3:**
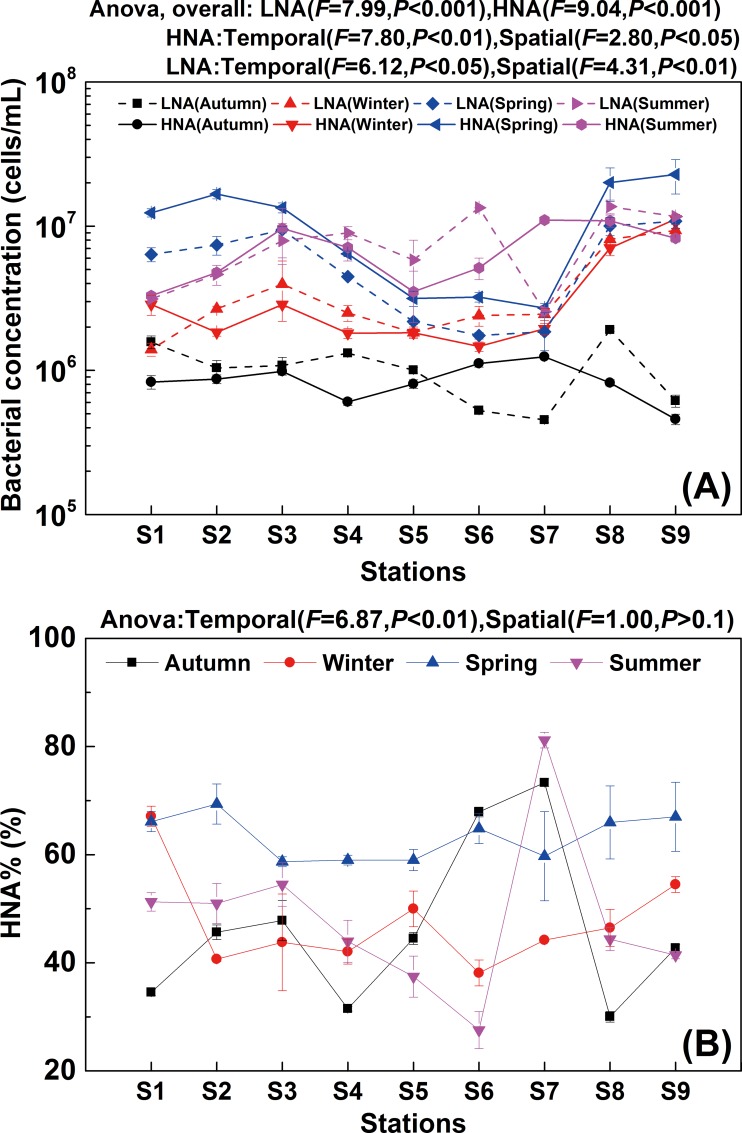
**Variations of LNA and HNA bacterial concentration (A) and percentage of HNA bacteria (B) along the Haihe River.** Error bars represent standard deviation of triplicate measurements.

As shown in the flow cytogram, LNA and HNA bacteria in the Haihe River could be clearly discriminated on the basis of their side scatter (SSC) and fluorescence intensity (FL1) in all four seasons (Fig 2). It showed that the bimodal distribution phenomenon based on nucleic acid content and cell size was commonly present in the planktonic bacteria from river ecosystems to oceans irrespective of seasonal shifts. Both LNA and HNA bacterial concentrations in spring and summer were significantly higher than those in winter and autumn (*P* < 0.05) (Fig 3). Although there were similar trends in temporal distributions between HNA and LNA bacteria, the one-way ANOVA analysis of HNA and LNA bacterial abundance showed that the variability in HNA (*F* = 9.04, *P* < 0.001) was greater than the variability in LNA (*F* = 7.99, *P* < 0.001) in different seasons (Fig 3A). Clear spatial variations were observed for both HNA and LNA bacteria along the river to the Bohai Sea (Fig 3). Meanwhile, the results showed that LNA bacteria had an equal share with HNA bacteria in all seasons except spring. In contrast, HNA bacteria dominated the community in spring (64.8%) (Fig 3). This is consistent with previous reports on variations of LNA bacterial abundance in marine environments. For example, Calvo-Diaz and Moran investigated seasonal dynamics of picoplankton in the central Cantabrian Sea (southern Bay of Biscay), where it was observed that HNA bacteria dominated the community in winter and spring (64%), while the proportion of LNA and HNA bacteria was almost equal in autumn and summer [26]. The results suggested that both HNA and LNA bacteria could potentially dominate the community and were important components in the microbial community in both freshwater and marine ecosystems.

**Fig 4:**
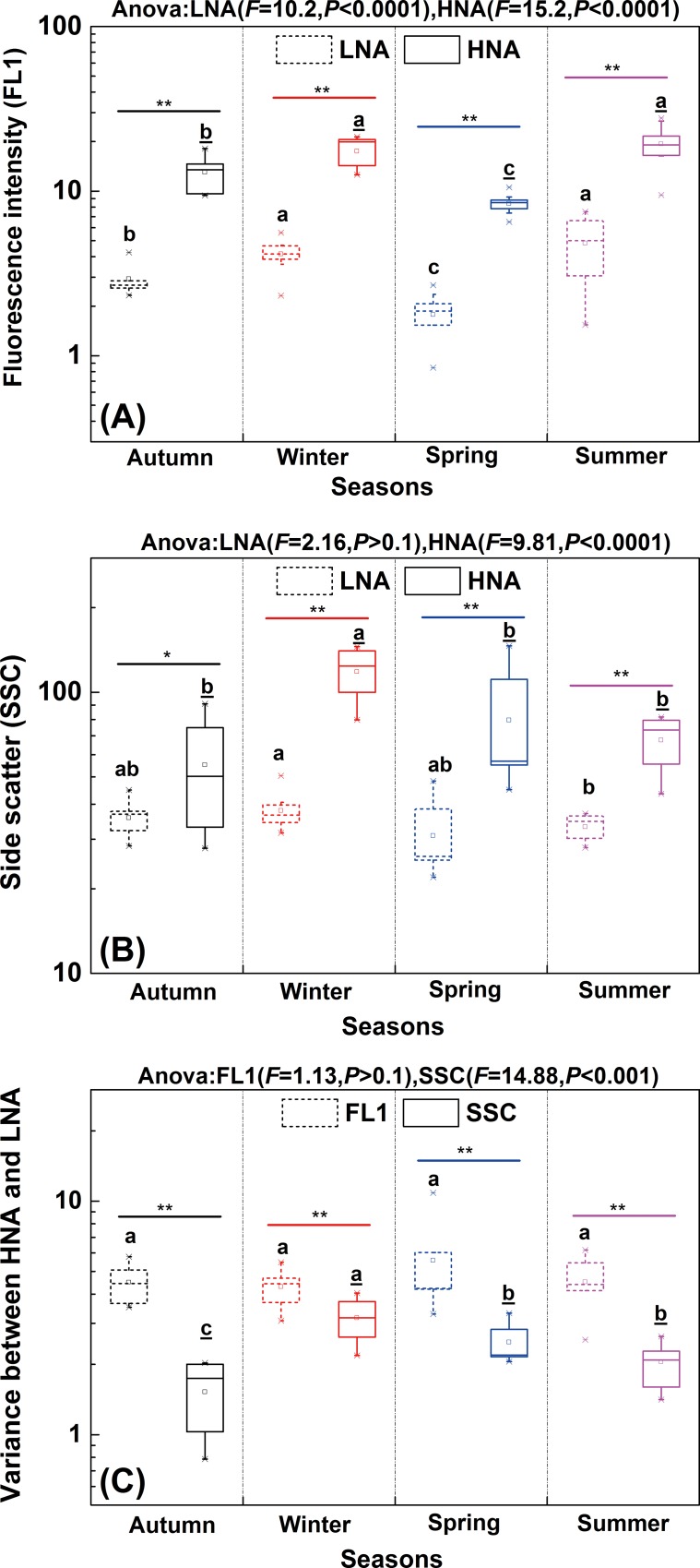
**Temporal changes of fluorescence intensity (FL1) (A), side scatter (SSC) (B) and variance between LNA and HNA of FL1 and SSC (C) in the Haihe River.** The box represents the range from 25% to 75% percentiles, whisker lines represent the outlier percentiles and the middle line in the box shows the median value of all data points. X-marks and square dots represent the outlier and mean values, respectively. Letters with and without underline represent seasonal differences of HNA and LNA bacteria respectively. Different lowercase letters indicate significant difference of 0.05. The symbols "**" and "*" indicate significant differences of 0.01 and 0.05, respectively.

With respect to flow cytometric characteristics, both green fluorescence intensity (FL1) (Pearson’s *R* = 0.850, *P* < 0.01) and side scatter (SSC) (Pearson’s *R* = 0.600, *P* < 0.01) of HNA and LNA were significantly correlated along the river. In comparison, no significant correlation was found between the FL1 and SSC within each subgroup (HNA: Pearson’s *R* = 0.371, *P* < 0.05, LNA: Pearson’s *P* > 0.1). In terms of temporal variation, both FL1 and SSC of HNA as well as FL1 of LNA showed significant changes between seasons (Fig 4A and 4B). Meanwhile, similar to the abundance variation, the variabilities of FL1 (*F* = 15.2, *P* < 0.0001) and SSC (*F* = 9.81, *P* < 0.0001) in HNA were greater than that in LNA (FL1: *F* = 10.2, *P* < 0.0001; SSC: *F* = 2.16, *P* > 0.1). In addition, based on the differences in cytometric parameters (FL1 and SSC) between HNA and LNA, the derived variable (FL1_HNA_/FL1_LNA_ and SSC_HNA_/SSC_LNA_) was used to figure out the relative distances between HNA and LNA in FCM. Such ratios could complementally represent variance within these two subgroups on FL1 and SSC, then further explain what and how different factors affect these variations [27]. The results showed that the relative distance of FL1 between HNA and LNA (median(min-max): 4.4(2.5–10.9)) was significantly higher than that of SSC (2.2(0.8–4.0)) (*P* < 0.01) in each season (Fig 4C). Furthermore, the relative distance of SSC between HNA and LNA showed significant temporal variation (*F* = 14.88, *P* < 0.001) (Fig 4C). The reason may lie in the seasonal variation of HNA and LNA bacterial activity which would result in the seasonal changes in cell size [28]. It was reported that the cell size of LNA bacteria became larger than that of HNA bacteria in the NW Mediterranean in March [21]. In contrast, no significant temporal variation was observed for the relative distance of FL1 (*F* = 1.13, *P >* 0.1) (Fig 4C). The different variability of FL1 and SSC suggested these two cytometric parameters would be related to different factors.

### Partition of driving factors on LNA and HNA bacteria

**Fig 5:**
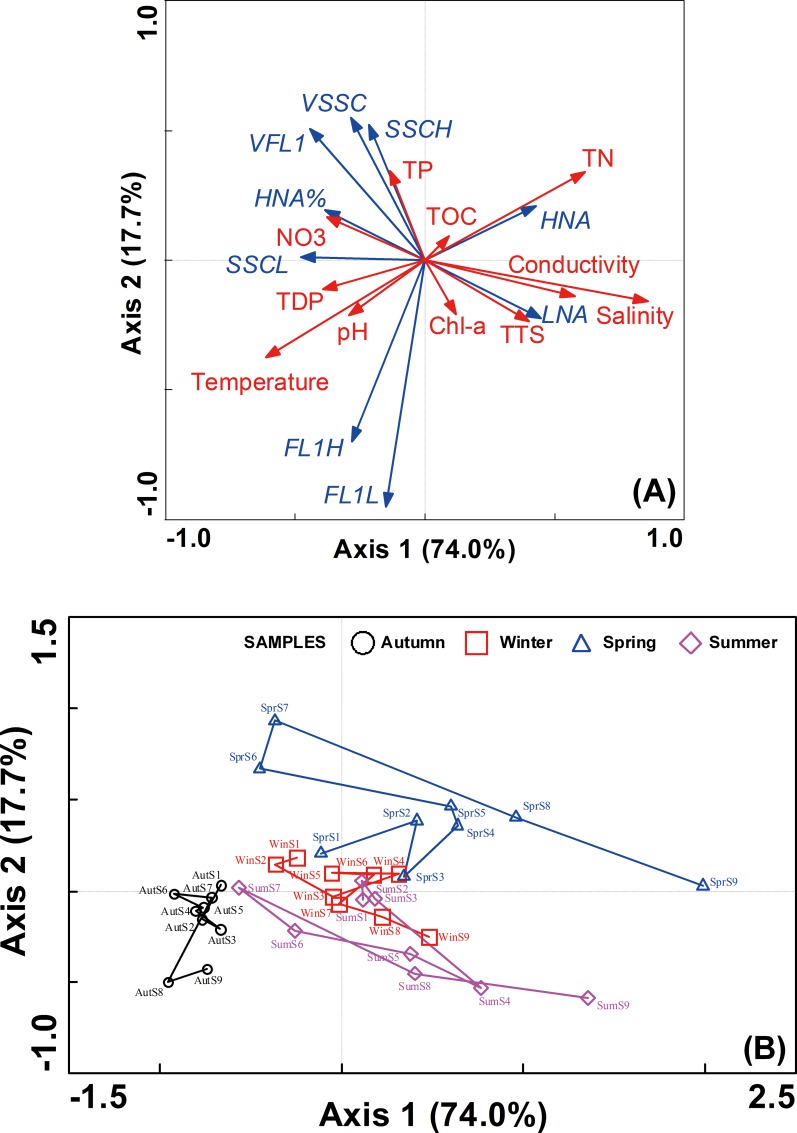
Redundancy analysis of LNA and HNA bacterial characteristic parameters with environmental factors in the Haihe River. (A) Biplots between characteristics of LNA\HNA and environmental variables, (B) Samples ordination. Abbreviations: TSS, total suspended solids; TN, total nitrogen; NO_3_, nitrate; TP, total phosphorus; TDP, total dissolved phosphorus; TOC, total organic carbon; Chl-a, chlorophyll-α; FL1L and FL1H, FL1 of LNA and HNA bacteria, SSCL and SSCH, SSC of LNA and HNA bacteria; VFL1, FL1_HNA_/FL1_LNA_; VSSC, SSC_HNA_/SSC_LNA_; LNA and HNA, the LNA and HNA bacterial concentration, HNA%, the percentage of HNA in total bacterial concentration. Samples were connected with lines according to location along with the Haihe River in subgraph B.

Redundancy analysis (RDA) was conducted to estimate how environmental variables influenced LNA and HNA bacteria (Fig 5). The Monte Carlo test showed that RDA axes 1 and 2 were significant to elucidate the correlations between bacterial properties and environmental variables (*F* = 18.26, *P* = 0.004). RDA revealed that salinity was the main driving factor for LNA and HNA bacteria distribution patterns, accounting for 20% variance (*F* = 8.26, *P* < 0.01), followed by temperature (explain 11% variance, *F* = 5.51, *P* < 0.01), conductivity (explain 8% variance, *F* = 4.10, *P* < 0.05), total phosphorus (explain 6% variance, *F* = 3.64, *P* < 0.05) and total suspended solids (explain 6% variance, *F* = 3.27, *P* < 0.05) (Fig 5A). The salinity primarily controlled the variation of LNA and HNA in the Haihe River, which indicated the geographical gradient along freshwater river to oceans have considerable influence on the distribution of these two subgroups. While most samples were seasonally clustered together, samples from S8 and S9 were separated from the others in each season; station S8 is close to the estuary and S9 is located in the estuary of Bohai Sea (the salinity was higher than other stations). The changes in temperature reflect the seasonal dynamics and also remarkably affect the characteristics of LNA and HNA, which coincide with the significant temporal variability on the abundance and cytometric parameters (Figs 3 and 4). Overall, these two major factors shaped the variation of LNA and HNA (Fig 5B).

It has been reported that HNA bacteria tend to grow in eutrophic and mesotrophic environments, while LNA bacteria reside in oligotrophic environments [29–31]. Our results showed that HNA and LNA bacterial abundance was significantly correlated to different environmental variables (Fig 5A). Specifically, LNA was negatively related to nitrate (NO_3_) and positively related to total suspended solids (TSS), while HNA was significantly related to total nitrogen (TN). A similar pattern between FL1 and SSC was observed, where the ordination of both cytometric parameters were divided in Fig 5A. The results revealed that the effects of geographical distance and temporal changes, which were characterized as salinity and temperature gradient respectively in RDA, were stronger than the effects of nutrient controls (e.g. TOC, TN and TP) on the LNA and HNA distribution (Fig 5A). The results are consistent with previous studies that seasonal changes in environmental variables have a more significant effect on microbial community patterns than trophic interactions [17,32]. Contemporary contingencies, e.g. local climate events, could drive the changes of biotic and abiotic factors on short time scales, then affects the microbial community dynamics [33–35].

**Fig 6:**
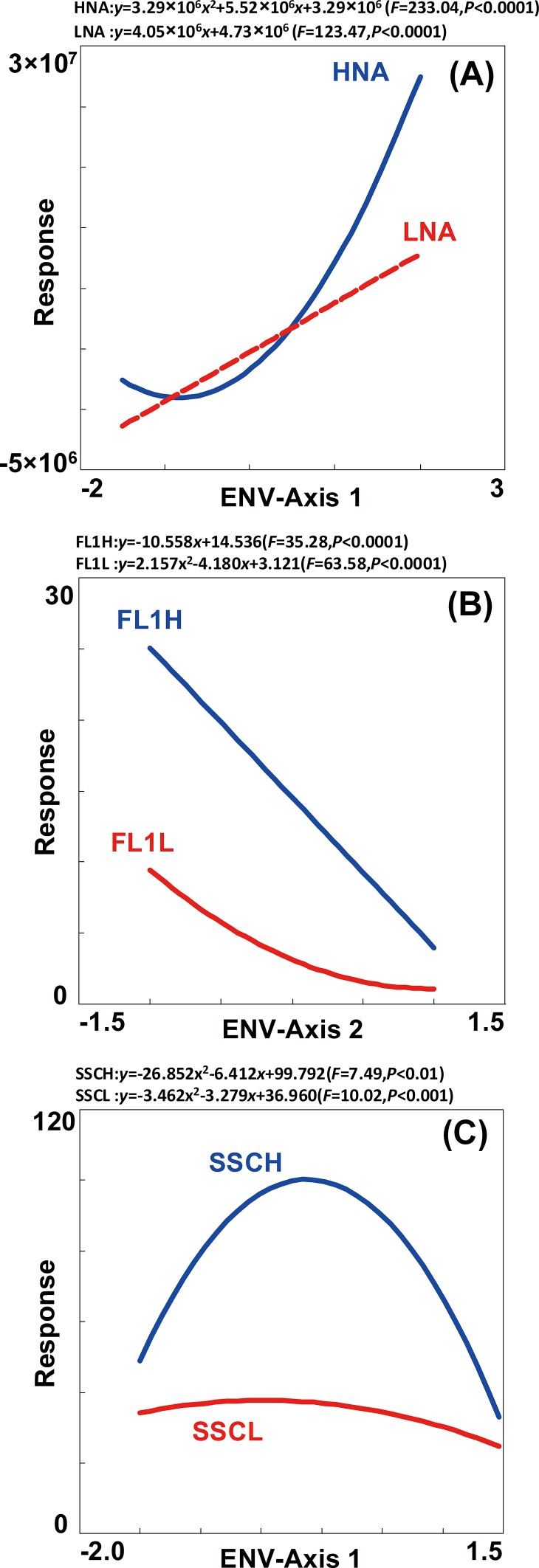
**Response of the abundance (A), FL1 (B) and SSC (C) of LNA and HNA bacteria to environmental ordination axis in RDA.** GLM and GAM were selected to fit the response analysis in a stepwise manner, as well environmental explanatory ordination axis 1 and 2 in RDA analysis, where only explanatory axis be of significance was drawn in the analysis processes.

Furthermore, response analysis was performed to graphically compare the differentiation on the changes of LNA and HNA bacterial abundance and cytometric parameters (FL1 and SSC). The response of HNA showed more variation than that of LNA (Fig 6A), which indicated the changes in HNA abundance maybe more sensitive to environmental variation in comparison to LNA. Meanwhile, response analysis showed that the variations of FL1 and SSC significantly correlated to axis 2 (Fig 6B) and axis 1 (Fig 6C), respectively. Different variation was observed in SSC and FL1 within and between LNA and HNA. The results demonstrated that these two cytometric parameters represented dissimilar characteristics in bacterial cells and could be regulated differently [27]. The dissimilarity along gradients within an ecosystem suggested that LNA and HNA bacteria might play different ecological roles.

## Conclusions

In summary, LNA and HNA bacteria make similar contributions to the total microbial abundance in an exorheic river on both spatial and temporal scale. The variability in HNA bacterial abundance and flow cytometric characteristics was greater than that of LNA bacteria. Meanwhile, the relative distance of SSC between HNA and LNA showed more variability than that of FL1. The present study demonstrated that the effects of geographical distance (salinity gradient along river to ocean) and temporal changes (temperature variation by seasonal succession) were stronger than the effects of nutrient conditions on the variations of LNA and HNA. Furthermore, the distribution of LNA and HNA bacteria, including the abundance and flow cytometric characteristics, were under the control of different environmental variables. The heterogeneity between LNA and HNA suggested those two subgroups may play different niche ecological roles in the microbial loop of aquatic ecosystems.
